# The Characterization of Structure and Prediction for Aquaporin in Tumour Progression by Machine Learning

**DOI:** 10.3389/fcell.2022.845622

**Published:** 2022-02-01

**Authors:** Zheng Chen, Shihu Jiao, Da Zhao, Quan Zou, Lei Xu, Lijun Zhang, Xi Su

**Affiliations:** ^1^ School of Applied Chemistry and Biological Technology, Shenzhen Polytechnic, Shenzhen, China; ^2^ Institute of Fundamental and Frontier Sciences, University of Electronic Science and Technology of China, Chengdu, China; ^3^ Yangtze Delta Region Institute (Quzhou), University of Electronic Science and Technology of China, Quzhou, China; ^4^ School of Electronic and Communication Engineering, Shenzhen Polytechnic, Shenzhen, China; ^5^ Foshan Maternal and Child Health Hospital, Foshan, China

**Keywords:** cancer, random forest, anova, 3D structure, machine learning

## Abstract

Recurrence and new cases of cancer constitute a challenging human health problem. Aquaporins (AQPs) can be expressed in many types of tumours, including the brain, breast, pancreas, colon, skin, ovaries, and lungs, and the histological grade of cancer is positively correlated with AQP expression. Therefore, the identification of aquaporins is an area to explore. Computational tools play an important role in aquaporin identification. In this research, we propose reliable, accurate and automated sequence predictor iAQPs-RF to identify AQPs. In this study, the feature extraction method was 188D (global protein sequence descriptor, GPSD). Six common classifiers, including random forest (RF), NaiveBayes (NB), support vector machine (SVM), XGBoost, logistic regression (LR) and decision tree (DT), were used for AQP classification. The classification results show that the random forest (RF) algorithm is the most suitable machine learning algorithm, and the accuracy was 97.689%. Analysis of Variance (ANOVA) was used to analyse these characteristics. Feature rank based on the ANOVA method and IFS strategy was applied to search for the optimal features. The classification results suggest that the 26th feature (neutral/hydrophobic) and 21st feature (hydrophobic) are the two most powerful and informative features that distinguish AQPs from non-AQPs. Previous studies reported that plasma membrane proteins have hydrophobic characteristics. Aquaporin subcellular localization prediction showed that all aquaporins were plasma membrane proteins with highly conserved transmembrane structures. In addition, the 3D structure of aquaporins was consistent with the localization results. Therefore, these studies confirmed that aquaporins possess hydrophobic properties. Although aquaporins are highly conserved transmembrane structures, the phylogenetic tree shows the diversity of aquaporins during evolution. The PCA showed that positive and negative samples were well separated by 54D features, indicating that the 54D feature can effectively classify aquaporins. The online prediction server is accessible at http://lab.malab.cn/∼acy/iAQP.

## Introduction

Water, as one of the most widely existing molecules, is the basic requirement for the development of organisms. Aquaporins (AQPs) are a large and evolutionarily conserved family of proteins that facilitate water absorption and flow across cytoplasmic compartments and cell membranes in microorganisms, animals, and plants. From a previous study, aquaporins, as water channel proteins, not only take part in water molecule transport but also respond to other small molecule transport, such as glycerol, urea, ammonia, and CO_2_, which help those molecules cross cell membranes (Preston et al., 1992; Ma et al., 1997; Agre et al., 2002; Nielsen et al., 2002; Rojek et al., 2008). In the aquaporin family, some aquaporins are primarily water selective, such as AQP1, AQP2, AQP4, AQP5 and AQP8, while other parts of the aquaporins, such as AQP3, AQP7, AQP9, and AQP10, transport water, glycerol and other small solutes (Verkman, 2005). Aquaporins are small highly conserved membrane proteins that can selectively promote water molecule transportation through the cell membrane. Aquaporins (AQPs), with a molecular weight of 28 kDa, were first found in the membrane of human red blood cells (Agre et al., 2002). AQPs usually exist as tetramers; when water passes through these narrow channels, the conformation of AQPs can decide whether water passes through the cell membrane.

AQPs not only act as channels to take part in water and small molecule transport but are also widely related to a variety of pathophysiological statuses in cells. Evidence of AQPs in cell proliferation has aroused great interest in the research of AQPs in tumour progression (Levin and Verkman, 2006; Zhang et al., 2010; Jung et al., 2011; Nakahigashi et al., 2011; Di Giusto et al., 2012; Direito et al., 2016; De Ieso and Yool, 2018). At present, AQPs can be expressed in many types of tumours, including in the brain (Maugeri et al., 2016; Lan et al., 2017), breast (Jung et al., 2011), pancreas (Arsenijevic et al., 2019), colon (Nagaraju et al., 2016), skin (Hara-Chikuma and Verkman, 2008a), ovaries (Kasa et al., 2019) and lung (Chae et al., 2008). There was a positive correlation between the histological tumour grade and AQP expression, such as the expression of AQP4 in diffuse astrocytoma (Saadoun et al., 2002a; Kröger et al., 2004).

For colorectal cancer, the expression of AQP8 decreased (Fischer et al., 2001), while that of AQP1, AQP3 and AQP5 increased (Moon et al., 2003), indicating that AQPs can be expressed in tumours in humans. In general, AQP expression is upregulated in tumours. Therefore, many studies speculate that aquaporins allow water to penetrate, resulting in rapid tumour mass formation. In astrocytomas, the expression level of AQP4 is related to the amount of oedema but not to survival status (Saadoun et al., 2002a; Warth et al., 2007). Recent studies have indicated that AQPs, as prognostic markers, have a potential role in tumour-associated oedema because they participate in angiogenesis, tumour cell migration and proliferation (Saadoun et al., 2005a; Saadoun et al., 2005b; Hara-Chikuma and Verkman, 2006; Auguste et al., 2007; Hara-Chikuma and Verkman, 2008b). AQP1, AQP4 and AQP9 are expressed in brain tumours, and AQP4 expression increases with the severity of brain oedema (Saadoun et al., 2002a; Ding et al., 2010; Wang and Owler, 2011; Ding et al., 2013; Maugeri et al., 2016; Lan et al., 2017). In brain, lung, prostate and colon tumours, AQP1 with high expression participates in cell migration and tumour angiogenesis (Saadoun et al., 2002b; Saadoun et al., 2005b; Mobasheri et al., 2005; Kang et al., 2008). AQP3 has increased expression in ESCA, COAD, LUAD and LIHC (Marlar et al., 2017). AQP3 knockout mice can inhibit the development of skin tumours, and tumorigenesis can utilize ATP produced by AQP3-mediated glycerol transport (Hara-Chikuma and Verkman, 2008a). AQP5 is also related to the migration, metastasis, and poor prognosis of cancer cells in BRCA (breast cancer) (Jung et al., 2011; Lee et al., 2014; Jensen et al., 2016). AQP5-regulating miRNAs inhibit BRCA cell migration through exosome-mediated delivery (Park et al., 2020). Under exosome-mediated delivery, AQP5-regulated miRNAs inhibit BRCA cell migration (Park et al., 2020).

Aquaporins play a role in the development and prognosis of various cancers, so the machine learning recognition method of aquaporins is also one of the hot spots in cancer research. Machine learning methods are applied to establish a novel and efficient classification model of aquaporins and are helpful to accelerate the recognition of aquaporins. The amino acid sequence composition of the protein is considered to be a sequence feature of the protein (Tyagi et al., 2013).

There are two methods for protein classification methods, as follows: one is based on protein sequence information (Liu et al., 2020a; Zhang et al., 2021), and the other is based on protein structure features (Liu et al., 2019; Cai et al., 2020). The sequence-based protein classification method extracts features by using the amino acid composition, amino acid number and other sequence information of the protein sequence (Liu et al., 2014). These methods are efficient and useful in predicting a large number of protein sequence datasets (Lou et al., 2014). At present, there are various studies on the classification of protein sequences, such as using logistic regression and support vector machine (SVM) methods to predict DNA binding proteins (Shen and Zou, 2020; Liu et al., 2021a) by considering amino acid proportions, amino acid compositions, amino acid spatial asymmetric distributions and biological coding characteristics of evolutionary information (Szilágyi and Skolnick, 2006; Kumar et al., 2007). The protein classification method based on protein structure identifies proteins by using structure and sequence information (Liu et al., 2014). Previous studies have focused on positive electrostatic potential, protein surface, overall charge and positive patches (Shanahan et al., 2004; Bhardwaj et al., 2005), which have achieved excellent results. Under certain conditions, the prediction accuracy of three protein motifs (helix turning helix, helix hairpin helix, and helix loop helix) is 91.1%, which indicates that this method is efficient for protein determination (Cai et al., 2009).

**FIGURE 1:**
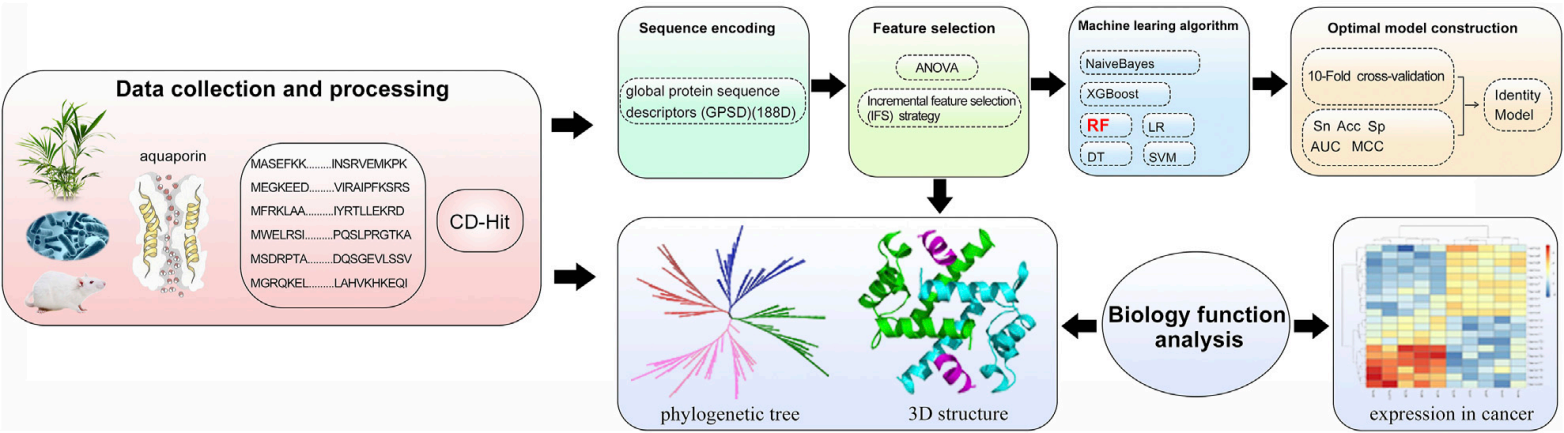
The whole framework of the method iAQPs-RF to identify the aquaporins.

In our work, to promote the rapid application of AQPs in cancer treatment, a powerful sequence-based analysis method to distinguish the AQPs and cross validation was applied for results demonstration (Figure 1). It is important to develop an effective model to predict AQPs. We propose a sequence-based AQP prediction model that performs stably on various classifiers. The AQP classification model uses the 188D feature extraction method, applies ANOVA to reduce the dimensionality, and uses different algorithms to optimize the AQP classification model. 188D is a characteristic of the frequency of continuous amino acid residues in proteins. ANOVA is used to prune features without affecting the accuracy of the predictor.

## Materials and Methods

### Dataset

A high-quality dataset is essential for reliable and accurate predictor building (Su et al., 2021). Aquaporin was taken as the positive sample, and the protein sequence was collected from the protein database of the UniProt website (https://www.uniprot.org/) (Chen et al., 2016). Negative samples such as nonaquaporins were extracted from the Pfam database (http://pfam.xfam.org/). To ensure the reliability of the aquaporin dataset, we applied the following criteria to optimize the data: first, the sequences annotated as “prediction” were eliminated; second, we deleted the sequences of other protein fragments; through screening steps, 239 aquaporin sequences and 10,713 nonaquaporin sequences were obtained; third, the CD-HIT program (Fu et al., 2012) was used to eliminate redundant sequences and to avoid overestimating the prediction model (Zou et al., 2020). The cut-off of sequence identity is set to 90%. Finally, 151 aquaporins and 8,994 nonaquaporins were obtained to form the final dataset.

### Features Extraction

One of the main factors for the performance accuracy of the prediction model is the quality of sample feature extraction. The prediction of the protein model mainly depends on the coding strategy of the protein sequence. According to the coding strategy of the protein sequence, the amino acid sequence can be transformed into a numerical vector (Liu et al., 2019; Muhammod et al., 2019; Zhu et al., 2019; Chen et al., 2020; Fu et al., 2020; Tang et al., 2020; Wang et al., 2020; Shao et al., 2021). In this paper, the global protein sequence descriptor (GPSD) method was used to represent the amino acid sequence. Global protein sequence descriptor (GPSD), known as 188 days method. This method mainly converts the sequence into a numerical vector according to the amino acid properties in the protein sequence and generates 188 features. These 188D features contain the information and properties of amino acid sequences [48,49]. According to the description of the GPSD method, the 188D features can be divided into two parts. The first part is the composition of amino acids. The first 20D features were obtained by calculating the frequency of amino acids in the protein sequence. The second part is to calculate the physicochemical properties of amino acids, which constitute 168 characteristics. Previous studies have provided detailed information on the eight physicochemical properties of amino acids (Lin et al., 2013; Liu et al., 2018; Li et al., 2019a). The protein sequence was encoded by CTD (C: composition, t: transition, D: distribution) mode to generate 21D features. Three groups were generated for 20 amino acids for each property. C is the occurrence frequencies (1 × 3D = 3D). T is the transition frequency (1 × 3D = 3D). D is the first, 25, 50, 75% and last position of a certain group in the peptide sequence (5 × 3 = 15D). Therefore, 8 * (3 + 3 + 15) = 168 features were produced for the CTD model.

### Classifier

To find the most suitable machine learning algorithm, six commonly used classifiers are applied, including random forest (RF) (Ru et al., 2019), NaiveBayes, support vector machine (SVM) (Wei et al., 2018a; Dao et al., 2020a; Dao et al., 2020b; Wei et al., 2020), XGBoost (Yu et al., 2021a; Yang et al., 2021), logistic regression (LR) and decision tree (DT) (Li et al., 2019b). These efficient machine learning algorithms are usually used for feature analysis.

### Feature Selection

For machine learning model building, features extracted from sequences always contain noise. A feature selection strategy to solve the information redundancy and overfitting problem can improve the feature representation ability (He et al., 2021). Analysis of variance (ANOVA) (Blanca et al., 2017; Wei et al., 2018b; Tang et al., 2018; Su et al., 2019a; Jung et al., 2019; Su et al., 2020; Liu et al., 2021b; Jin et al., 2021) has been used to analyse these characteristics and has been widely used in RNA, DNA and protein prediction. In this study, ANOVA is used to select the optimal features for model training. The feature subset with low redundancy is selected by ANOVA. We sort the original features based on the ANOVA feature sorting algorithm and apply the IFS strategy to search the optimal feature subset.

### Performance Standard

To evaluate the prediction accuracy of the model, the data of the following four formulas are usually used to solve the problem of classification prediction.

$$Acc=\frac{TP+TN}{\left(TP+TN+FP+FN\right)}$$

$$Sn=\frac{TP}{\left(TP+FN\right)}$$

$$Sp=\frac{TN}{\left(TN+FP\right)}$$

$$MCC=\frac{TP\times TN-FP\times FN}{\sqrt{\left(TP+FP\right)\left(TP+FN\right)\left(TN+FP\right)\left(TN+FN\right)}}$$

Accuracy (Acc), specificity (Sp), sensitivity (Sn) and Matthew correlation coefficient (MCC) were the commonly used evaluation parameters (Jiang et al., 2013; Wei et al., 2014; Wei et al., 2017a; Wei et al., 2017b; Wei et al., 2017c; Manavalan et al., 2019a; Manavalan et al., 2019b; Su et al., 2019b; Hong et al., 2019; Zeng et al., 2019; Zhang et al., 2020a; Liu et al., 2020b; Zeng et al., 2020; Yu et al., 2021b; Jin et al., 2021; Shao and Liu, 2021; Zhu et al., 2021). In the formulas, TP was the true positive number, TN was the true negative number, FP was the false-positive number and FN was the false negative number.

A receiver operating characteristic (ROC) curve was applied to study the prediction performance of the model. The area under the ROC curve (AUC) was used to assess the prediction performance of the model. AUC values of 0.5 and one represent random and perfect models, respectively (Zeng et al., 2017; Zeng et al., 2018; Dao et al., 2019; Feng et al., 2019; Lai et al., 2019; Lin et al., 2019; Zhu et al., 2019; Zhang et al., 2020b; Charoenkwan et al., 2020; Ding et al., 2020a; Ding et al., 2020b; Hasan et al., 2020; Huang et al., 2020; Jin et al., 2020; Li et al., 2020; Wang et al., 2020; Wu and Yu, 2021).

### Construction of 3D Structure for AQPs

To verify the localization of aquaporins, the website (http://www.csbio.sjtu.edu.cn/bioinf/Cell-PLoc-2/) of the protein localization website and transmembrane prediction website (https://www.novopro.cn/tools/tmhmm.html) were applied to predict the subcellular localization and transmembrane structure of aquaporins. At the same time, Phyre2 software (http://www.sbg.bio.ic.ac.uk/phyre2/html/page.cgi?id=index) was applied for the 3D structure prediction of aquaporins. The prediction results were visualized by PyMOL (version 2.5.1) software (https://pymol.org/2/).

### Construction of Aquaporins Phylogenetic Tree

The phylogenetic tree of aquaporins was constructed to analyse the evolutionary diversity of the protein. Aquaporin sequence alignment results were analysed by MAFFT online software (https://mafft.cbrc.jp/alignment/server/) and used to construct a phylogenetic tree using IQ-TREE software (multicore version 1.6.12). The best fitting model for the phylogenetic tree was LG + F + R6 (Kalyaanamoorthy et al., 2017). The ultrafast bootstrap method was used for phylogenetic assessment, and 1,000 replicates per method were chosen in this work (Guindon et al., 2010; Minh et al., 2013; Hoang et al., 2018). The tree file was visualized by the iTOL website (https://itol.embl.de/).

## Experiment

### Performance of Features Based on the 188-Dimensional Method (GPSD)

To select the best classifier for the AQP sequences, six widely used machine learning classifiers were employed to classify the features of AQP sequences extracted by the 188-dimensional method (GPSD). For feature extraction by the 188-dimensional method (GPSD), we applied different ratios for the number of positive and negative samples (1:1, 1:2, 1:3, 1:4, 1:5, and 151:8,994), and the results were classified by six machine learning classifiers (XGBoost, Naivebayes, LR, decision tree, RF and SVM). The results of all classifiers in the tenfold cross-validation were compared, and the comparison results are shown in Table 1.

**TABLE 1:** Preliminary results of different feature descriptors using different classifiers.

188D_P: N = 1:1	Sn	Sp	Acc	MCC	AUROC
XGBoost	98	96.04	97.033	0.9416	0.9949
NaiveBayes	96.666	96.041	96.366	0.9279	0.9763
LR	97.999	94.748	96.377	0.9289	0.9857
DecisionTree	95.999	95.374	95.689	0.9155	0.9569
RF	98.666	96.707	97.689	0.9544	0.9987
SVM	95.332	96.04	95.7	0.9153	0.9917
188D_P: N = 1:2	Sn	Sp	Acc	MCC	AUROC
NaiveBayes	98	97.667	97.793	0.9531	0.9765
SVM	92.75	98.334	96.471	0.9224	0.9965
LR	96.083	98.001	97.359	0.9425	0.9958
RF	97.332	98.344	98.012	0.9564	0.9978
XGBoost	96.708	96.677	96.682	0.9284	0.9954
DecisionTree	92.041	93.687	93.141	0.8518	0.9286
188D_P: N = 1:3	Sn	Sp	Acc	MCC	AUROC
NaiveBayes	97.333	96.015	96.357	0.9102	0.9765
SVM	90.75	99.334	97.181	0.9244	0.9979
LR	95.417	98.445	97.682	0.9394	0.9952
RF	96.666	98.455	98.013	0.948	0.9979
XGBoost	95.374	98.011	97.343	0.9306	0.995
DecisionTree	93.999	96.697	96.024	0.8974	0.9535
188D_P: N = 1:4	Sn	Sp	Acc	MCC	AUROC
NaiveBayes	97.333	97.18	97.22	0.9206	0.9771
SVM	92.083	99.005	97.617	0.9256	0.9946
LR	93.457	97.844	96.953	0.9074	0.9942
RF	94.709	99.166	98.274	0.9461	0.9967
XGBoost	95.333	98.668	98.007	0.9391	0.9958
DecisionTree	92.708	98.841	97.614	0.9248	0.9578
188D_P: N = 1:5	Sn	Sp	Acc	MCC	AUROC
NaiveBayes	96.666	97.084	97.019	0.9022	0.9773
SVM	92.083	99.205	98.015	0.9292	0.9954
LR	93.999	98.143	97.46	0.9136	0.996
RF	94.667	99.338	98.562	0.9486	0.9975
XGBoost	95.333	98.94	98.341	0.9414	0.9963
DecisionTree	91.374	98.01	96.905	0.8924	0.9469
188D_P: N (151:8,994)	Sn	Sp	Acc	MCC	AUROC
XGBoost	86.084	99.934	99.703	0.9062	0.9989
NaiveBayes	96.666	97.977	97.955	0.6522	0.9793
LR	84.082	99.635	99.374	0.8158	0.9975
DecisionTree	72.208	99.365	98.918	0.6827	0.8579
RF	82.75	99.912	99.626	0.879	0.995
SVM	31.167	100	98.866	0.5503	0.9916

The results of Table 1 show that the different proportions of positive and negative samples indicated that P: N = 1:1 was the best ratio for the following analysis. Although the values of 1:2, 1:3, 1:4, 1:5 and 151:8,989 have higher values in SP and ACC, the values of Sn, MCC and AUROC are lower compared with P: N = 1:1. The increase in negative samples causes data imbalance and overfitting of the model. Therefore, the positive and negative sample ratio column of P: N = 1:1 is selected for model building.

**FIGURE 2:**
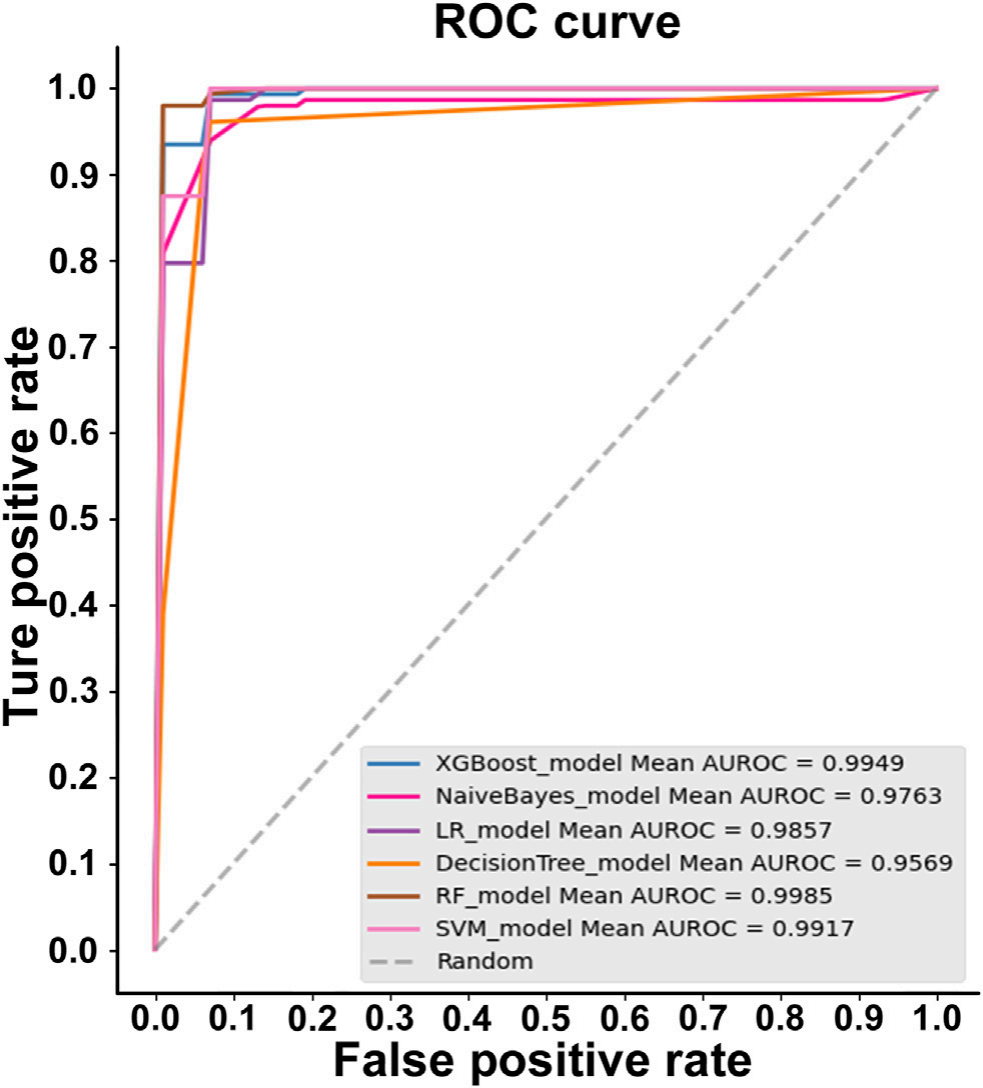
ROC curves for the best performing feature with different classifiers.

For the AQP sequences (P: N = 1:1), random forest (RF) was the best algorithm, with the highest accuracy for the features extracted by the 188-dimensional method (GPSD) (AUC = 0.9987, Acc = 97.689%, MCC = 0.9544, Sn = 98.666%, Sp = 96.707%). XGBoost is the second algorithm with a slightly lower accuracy (AUC = 0.9949, Acc = 97.033%, MCC = 0.9416, Sn = 98%, Sp = 96.04%) compared with the random forest (RF) algorithm. The NaiveBayes, LR, DecisionTree and SVM algorithms have similar accuracies lower than the random forest (RF) algorithm for AQP sequence classification based on the 188-dimensional method (GPSD). The results in Figure 2 indicated that RF was the best classifier with an accuracy of 0.9985, while the other classifiers of XGBoost, Naivebayes, LR, decision tree and SVM had accuracies of 0.9949, 0.9763, 0.9857, 0.9569 and 0.9917, respectively. In this study, six widely used classifiers are used for classification. The ROC of the RF classifier is 0.9985, which is relatively high. In general, regarding the evaluated accuracy of the AUC, Acc and MCC values, RF had the best performance in the AQP sequence classification results and was selected as the best classifier for model building.

### Effect of Feature Selection Technologies

**FIGURE 3:**
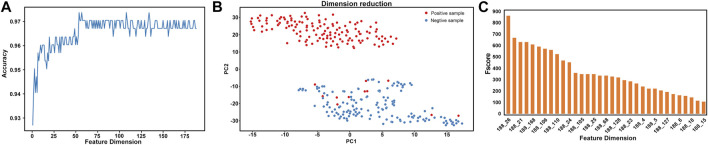
Two-step feature selection result display **(A)** 10-fold CV and independent test accuracy of the RF classifier with the feature number varied **(B)** dimension reduction results based on the PCA method for the original data with a total of 188 dimensions **(C)** feature ranking of the F-score method obtained by ANOVA for the data with 188 features.

However, there are redundant or noisy features among the features extracted by the 188D method, which will affect the stability of the model. To overcome these effects, we use the ANOVA feature selection method to optimize these features. The optimized classification results of the feature selection method based on ANOVA are shown in Table 2. In addition, the optimal feature 54D is selected by combining ANOVA with an incremental feature selection (IFS) strategy, as shown in Figure 3A. The comparison results show that the accuracy of the optimal feature selected (ACC = 97.689) is slightly higher than that of the original feature (ACC = 97.356) (Table 2). Therefore, the ANOVA feature selection method was selected for feature optimization.

**TABLE 2:** ANOVA feature selection methods based on random forest.

ANOVA	Sn	Sp	Acc	MCC	AUROC
188D	98.666	96.04	97.356	0.9479	0.9991
ANOVA_ 54D	98.666	96.707	97.689	0.9544	0.997

The PCA method was used to visually analyse the optimal feature (54D) after feature selection by the feature selection method (Figure 3B). Figure 3B indicates that positive and negative samples can almost be separated in the two-dimensional visualization diagram, which indicates that the 54D feature can effectively classify AQP proteins.

### Feature Distribution Analysis

In this study, we performed feature analysis after feature selection. By analysing these 188D features, we determine the attribute information contained in these features. The results of feature analysis are shown in Figure 3C. According to the best feature analysis of the F-score value obtained by ANOVA, the features with an F-score value greater than 100 have a greater contribution to the classification. It can be seen from the figure that among the 188D features, the first is the 26th dimension feature, which is neutral/hydrophobic, followed by the 21st dimension feature, which is hydrophobic. The 26th dimension feature (neutral/hydrophobic) and 21st dimension feature (hydrophobic) signs showed that AQPs contained hydrophobic amino acids, which may be associated with the structural and functional properties of AQPs.

### Structure Analysis of AQPs

Through feature selection, we know that hydrophobic features (the 26th dimension feature and 21st dimension feature) are the most significant features and make a great contribution to classification. Therefore, we analysed the protein localization of the AQP protein sequence, and the results showed that all AQP proteins were located on the cell membrane (Supplement Table 1). Cells are distinguished by a thin membrane. The core of the membrane is hydrophobic, which means it repels water. Many signals and nutrients cannot pass through the membrane itself but can pass through proteins across the membrane. Membrane proteins are essential for living cells, and plasma membrane proteins also have properties such as hydrophobicity, low solubility and low abundance. Therefore, the enrichment and classification extraction methods of soluble proteins cannot be used for plasma membrane proteins, mainly because the expression level of plasma membrane proteins in cells is very low, and they are highly hydrophobic in nature, which makes them easier to precipitate in aqueous solution and difficult to extract (Luche et al., 2003; Rawlings, 2016).

**FIGURE 4:**
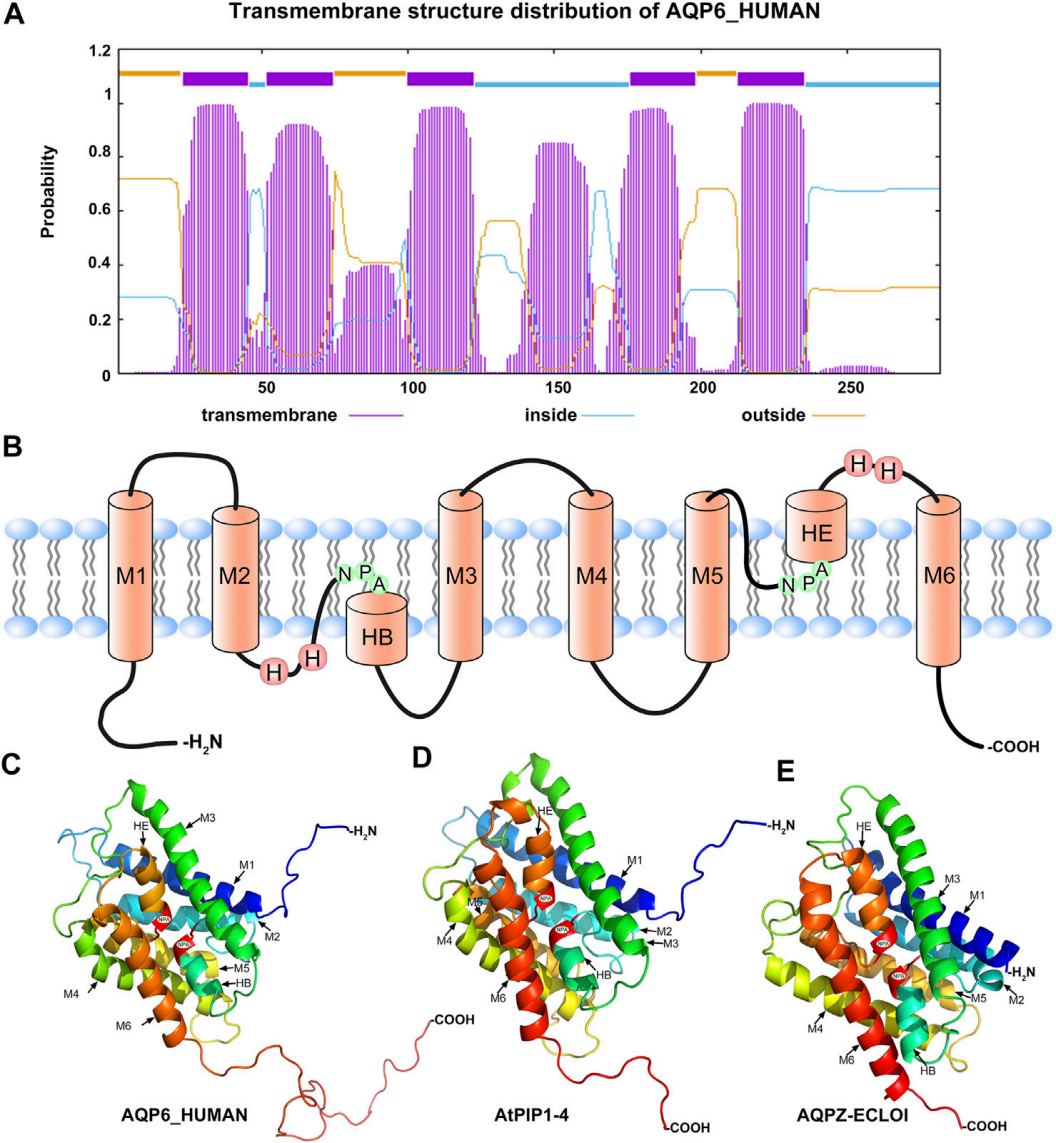
The structure of AQPs **(A)** The prediction distribution of the transmembrane structure for AQP6_HUMAN **(B)** model of the structure of an AQP showing the principal features of the protein, NPA: asparagine-proline-alanine motifs; M1-M6: the transmembrane structure **(C–E)** 3D constructure of AQP6_HUMAN, AtPIP1-4 and AQPZ-ECLOI.

The Phyre2 website was used to analyse the transmembrane structure of HmAQP7. Figure 2 shows that there are six α-helix transmembrane domains (Figure 4A): M1, M2, m3, M4, M5 and M6 (Figure 4B). A six-α-helix transmembrane domain forms a pore on the cell membrane to supply water molecules through the cell membrane. When the AQP protein folds, loops B (HB) and E (HE), which retain the lipophilic half helix, project to the protein molecular centre, making the highly conserved Asn-Pro-Asp (NPA) motif present the opposite direction, thus regulating the single file conductance of water and acting as a cation and proton exclusion filter (Figures 4B–E).

### Evolution and Diversity

**FIGURE 5:**
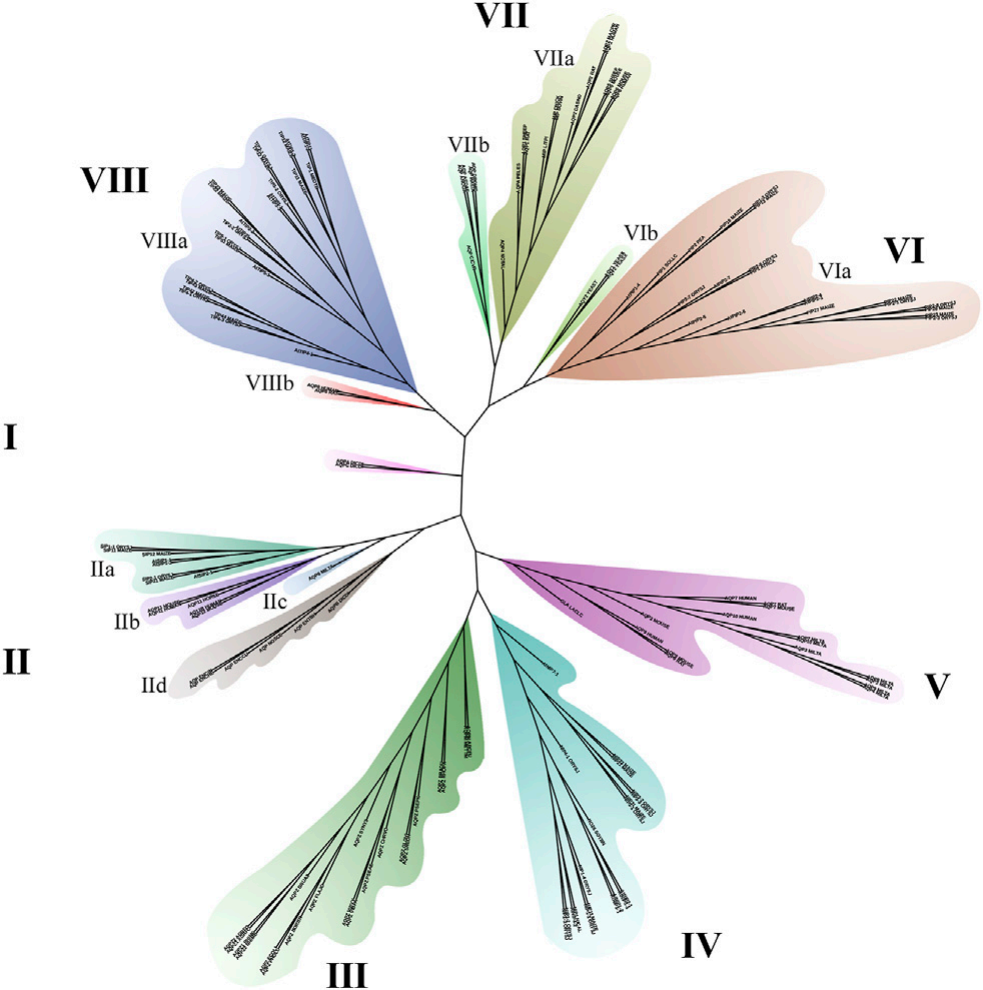
Phylogenetic analysis of positive AQP proteins.

Aquaporin is a conserved membrane protein that contains highly conserved NAP domains and α-helical transmembrane domains in bacteria (Figure 4E), plants (Figure 4D) and humans (Figure 4C). To better verify the phylogenetic and evolutionary relationship of AQPs, 151 AQP protein sequences containing human, mouse, insect, fungus and bacteria were applied to construct a phylogenetic tree (Figure 5).

The results indicated that the 151 AQP protein sequences were divided into eight groups (Figure 5). The length of branches indicates the genetic relationship of AQP sequences. Among them, group Ⅲ and group Ⅳ belong to plant and bacteria branches, respectively. Group Ⅱ is the most complex branch, including the aquaporins of fungi, bacteria and animals. Among them, the VIa and VIIIa branches are plant subfamilies. AQPs of the VIIa and VIIb subfamilies belong to animals and insects, respectively. Group V contains one bacterial AQPZ and 15 animal AQPs, of which 7 belong to *Tardigrade*.

### Expression of AQPs in Tumour Tissue

**FIGURE 6:**
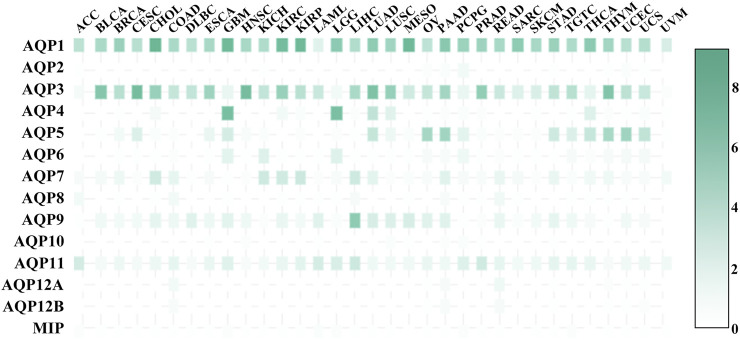
Expression of AQPs in 33 human tumours.

AQPs are considered to be important prognostic markers of cancers (Chow et al., 2020), so the expression of AQPs in cancer tissues is also crucial. Figure 6 shows the expression level of AQP transcripts in 33 tumour tissues. AQP1_HUMAN has a high expression level in all tumour tissues and plays an important role in tumour angiogenesis and endothelial cell migration (Saadoun et al., 2005b). AQP3_HUMAN is expressed in almost all tumour tissues except ACC, LGG, UVM and AQP3_HUMAN-mediated glycerol transport, which allows the production of ATP for tumorigenesis. AQP3_HUMAN knockout mice can be resistant to carcinogen induction skin tumours (Hara-Chikuma and Verkman, 2008a). AQP3_HUMAN and AQP5_HUMAN were also expressed in COAD (Moon et al., 2003), while AQP5_HUMAN expression in human COAD is related to cell proliferation and metastasis. In BRCA, AQP5_HUMAN overexpression is associated with (Jung et al., 2011; Lee et al., 2014; Jensen et al., 2016) migration and poor prognosis in BRCA patients. Consistently, AQP5_HUMAN regulates miRNA migration through exosome-mediated (Park et al., 2020) and inhibits BRCA cell migration. AQP2_HUMAN, AQP12A_HUMAN, AQP12B_HUMAN and MIP had low expression levels in 33 tumour tissues, AQP4_HUMAN was highly expressed in GBM and LGG, and AQP9_HUMAN was highly expressed in LIHC.

### Web Server Implementation

To facilitate the prediction of aquaporins, a user-friendly online server named iAQPs-RF is applied, which can be accessed from http://lab.malab.cn/∼acy/iAQP. The protein sequences (FASTA format) were identified to determine whether aquaporins or non-aquaporins use the web server by users. First, the FASTA format protein sequences are enterd or pasted in the left blank box and the submit button is clicked; finally, the results are displayed on the right box. If you want to restart a new task, a clear button or the resubmit button was clicked to clear the sequences in the input box. Finally, new query protein sequences were allowed to enter the input box. The home page provides links of the contact information of authors and relevant data to download.

## Conclusion

The accurate identification of aquaporins by iAQPs can greatly promote the prediction of aquaporins and research on tumour diseases. In this study, we used the GPSD method to extract protein sequence features and the optimal random forest algorithm to construct new computational aquaporin identifier iAQPs-RF. Combined with the feature selection technique ANOVA, 54 optimal features are selected to build the predictor. According to the F-score value obtained by ANOVA, the 26th dimension feature and 21st dimension feature are ranked as the first and second dimension features among the 188 days features, respectively, and these two features possess neutral/hydrophobic characteristics. These two dimensional features make a great contribution to the classification of aquaporins. At the same time, through the location and 3D structure prediction of aquaporins protein, although the protein divided into eight groups and has diversity in evolution, all the proteins belong to plasma membrane proteins, and the protein sequence contains six α-helix transmembrane domains. The membrane proteins are hydrophobic and contain many hydrophobic amino acids (Luche et al., 2003; Rawlings, 2016), so these results are consistent with aquaporin classification.

The best CV evaluation accuracy of iAQPs-RF was 97.689%. At the same time, a network server is established. iAQPs-RF are expected to be a robust and reliable tool for aquaporin identification. Future work will focus on exploring deep learning to improve the performance of the model.

## Data Availability

The original contributions presented in the study are included in the article/Supplementary Material, further inquiries can be directed to the corresponding authors.

## References

[B1] Agre P. King L. S. Yasui M. Guggino W. B. Ottersen O. P. Fujiyoshi Y. (2002). Aquaporin Water Channels - from Atomic Structure to Clinical Medicine. J. Physiol. 542, 3–16. 10.1113/jphysiol.2002.020818 12096044 PMC2290382

[B2] Arsenijevic T. Perret J. Van Laethem J.-L. Delporte C. (2019). Aquaporins Involvement in Pancreas Physiology and in Pancreatic Diseases. Ijms 20 (20), 5052. 10.3390/ijms20205052 PMC6834120 31614661

[B3] Auguste K. I. Jin S. Uchida K. Yan D. Manley G. T. Papadopoulos M. C. (2007). Greatly Impaired Migration of Implanted Aquaporin‐4‐deficient Astroglial Cells in Mouse Brain toward a Site of Injury. FASEB j. 21 (1), 108–116. 10.1096/fj.06-6848com 17135365

[B4] Bhardwaj N. Langlois R. E. Zhao G. Lu H. (2005). Kernel-based Machine Learning Protocol for Predicting DNA-Binding Proteins. Nucleic Acids Res. 33 (20), 6486–6493. 10.1093/nar/gki949 16284202 PMC1283538

[B5] Blanca M. J. Alarcón R. Arnau J. Bono R. Bendayan R. (2017). Non-normal Data: Is ANOVA Still a Valid Option? Psicothema 29 (4), 552–557. 10.7334/psicothema2016.383 29048317

[B6] Cai L. Wang L. Fu X. Xia C. Zeng X. Zou Q. (2020). ITP-pred: an Interpretable Method for Predicting, Therapeutic Peptides with Fused Features Low-Dimension Representation. Brief. Bioinform. 22, bbaa367. 10.1093/bib/bbaa367 33313672

[B7] Cai Y. He J. Li X. Lu L. Yang X. Feng K. (2009). A Novel Computational Approach to Predict Transcription Factor DNA Binding Preference. J. Proteome Res. 8 (2), 999–1003. 10.1021/pr800717y 19099508

[B8] Chae Y. K. Woo J. Kim M.-J. Kang S. K. Kim M. S. Lee J. (2008). Expression of Aquaporin 5 (AQP5) Promotes Tumor Invasion in Human Non Small Cell Lung Cancer. PLoS One 3 (5), e2162. 10.1371/journal.pone.0002162 18478076 PMC2364652

[B9] Charoenkwan P. Yana J. Schaduangrat N. Nantasenamat C. Hasan M. M. Shoombuatong W. (2020). iBitter-SCM: Identification and Characterization of Bitter Peptides Using a Scoring Card Method with Propensity Scores of Dipeptides. Genomics 112 (4), 2813–2822. 10.1016/j.ygeno.2020.03.019 32234434

[B10] Chen X.-X. Tang H. Li W.-C. Wu H. Chen W. Ding H. (2016). Identification of Bacterial Cell Wall Lyases via Pseudo Amino Acid Composition. Biomed. Res. Int. 2016, 1–8. 10.1155/2016/1654623 PMC4942628 27437396

[B11] Chen Z. Zhao P. Li F. Marquez-Lago T. T. Leier A. Revote J. (2020). iLearn: an Integrated Platform and Meta-Learner for Feature Engineering, Machine-Learning Analysis and Modeling of DNA, RNA and Protein Sequence Data. Brief. Bioinformatics 21 (3), 1047–1057. 10.1093/bib/bbz041 31067315

[B12] Chow P. H. Bowen J. Yool A. J. (2020). Combined Systematic Review and Transcriptomic Analyses of Mammalian Aquaporin Classes 1 to 10 as Biomarkers and Prognostic Indicators in Diverse Cancers. Cancers 12, 1911. 10.3390/cancers12071911 PMC7409285 32679804

[B13] Dao F.-Y. Lv H. Wang F. Feng C.-Q. Ding H. Chen W. (2019). Identify Origin of Replication in *Saccharomyces cerevisiae* Using Two-step Feature Selection Technique. Bioinformatics (Oxford, England) 35 (12), 2075–2083. 10.1093/bioinformatics/bty943 30428009

[B14] Dao F.-Y. Lv H. Yang Y.-H. Zulfiqar H. Gao H. Lin H. (2020). Computational Identification of N6-Methyladenosine Sites in Multiple Tissues of Mammals. Comput. Struct. Biotechnol. J. 18, 1084–1091. 10.1016/j.csbj.2020.04.015 32435427 PMC7229270

[B15] Dao F.-Y. Lv H. Zulfiqar H. Yang H. Su W. Gao H. (2020). A Computational Platform to Identify Origins of Replication Sites in Eukaryotes. Brief Bioinform 22, 1940–1950. 10.1093/bib/bbaa017 32065211

[B16] De Ieso M. L. Yool A. J. (2018). Mechanisms of Aquaporin-Facilitated Cancer Invasion and Metastasis. Front. Chem. 6, 135. 10.3389/fchem.2018.00135 29922644 PMC5996923

[B17] Di Giusto G. Flamenco P. Rivarola V. Fernández J. Melamud L. Ford P. (2012). Aquaporin 2-increased Renal Cell Proliferation Is Associated with Cell Volume Regulation. J. Cell. Biochem. 113 (12), 3721–3729. 10.1002/jcb.24246 22786728

[B18] Ding T. Gu F. Fu L. Ma Y.-J. (2010). Aquaporin-4 in Glioma Invasion and an Analysis of Molecular Mechanisms. J. Clin. Neurosci. 17 (11), 1359–1361. 10.1016/j.jocn.2010.02.014 20685122

[B19] Ding T. Zhou Y. Sun K. Jiang W. Li W. Liu X. (2013). Knockdown a Water Channel Protein, Aquaporin-4, Induced Glioblastoma Cell Apoptosis. PLoS One 8 (8), e66751. 10.1371/journal.pone.0066751 23950863 PMC3741385

[B20] Ding Y. Tang J. Guo F. (2020a). Identification of Drug-Target Interactions via Dual Laplacian Regularized Least Squares with Multiple Kernel Fusion. Knowledge-Based Syst. 204, 106254. 10.1016/j.knosys.2020.106254

[B21] Ding Y. Tang J. Guo F. (2020b). Identification of Drug-Target Interactions via Fuzzy Bipartite Local Model. Neural Comput. Applic 32, 10303–10319. 10.1007/s00521-019-04569-z

[B22] Direito I. Madeira A. Brito M. A. Soveral G. (2016). Aquaporin-5: from Structure to Function and Dysfunction in Cancer. Cell. Mol. Life Sci. 73 (8), 1623–1640. 10.1007/s00018-016-2142-0 26837927 PMC11108570

[B23] Feng C.-Q. Zhang Z.-Y. Zhu X.-J. Lin Y. Chen W. Tang H. (2019). iTerm-PseKNC: a Sequence-Based Tool for Predicting Bacterial Transcriptional Terminators. Bioinformatics (Oxford, England) 35 (9), 1469–1477. 10.1093/bioinformatics/bty827 30247625

[B24] Fischer H. Stenling R. Rubio C. Lindblom A. (2001). Differential Expression of Aquaporin 8 in Human Colonic Epithelial Cells and Colorectal Tumors. BMC Physiol. 1, 1. 10.1186/1472-6793-1-1 11231887 PMC32180

[B25] Fu L. Niu B. Zhu Z. Wu S. Li W. Cd-Hit (2012). CD-HIT: Accelerated for Clustering the Next-Generation Sequencing Data. Bioinformatics 28 (23), 3150–3152. 10.1093/bioinformatics/bts565 23060610 PMC3516142

[B26] Fu X. Cai L. Zeng X. Zou Q. (2020). StackCPPred: a Stacking and Pairwise Energy Content-Based Prediction of Cell-Penetrating Peptides and Their Uptake Efficiency. Bioinformatics 36 (10), 3028–3034. 10.1093/bioinformatics/btaa131 32105326

[B27] Guindon S. Dufayard J.-F. Lefort V. Anisimova M. Hordijk W. Gascuel O. (2010). New Algorithms and Methods to Estimate Maximum-Likelihood Phylogenies: Assessing the Performance of PhyML 3.0. Syst. Biol. 59 (3), 307–321. 10.1093/sysbio/syq010 20525638

[B28] Hara-Chikuma M. Verkman A. S. (2006). Aquaporin-1 Facilitates Epithelial Cell Migration in Kidney Proximal Tubule. Jasn 17 (1), 39–45. 10.1681/asn.2005080846 16319186

[B29] Hara-Chikuma M. Verkman A. S. (2008). Aquaporin-3 Facilitates Epidermal Cell Migration and Proliferation during Wound Healing. J. Mol. Med. 86 (2), 221–231. 10.1007/s00109-007-0272-4 17968524

[B30] Hara-Chikuma M. Verkman A. S. (2008). Prevention of Skin Tumorigenesis and Impairment of Epidermal Cell Proliferation by Targeted Aquaporin-3 Gene Disruption. Mol. Cell Biol 28 (1), 326–332. 10.1128/mcb.01482-07 17967887 PMC2223314

[B31] Hasan M. M. Schaduangrat N. Basith S. Lee G. Shoombuatong W. Manavalan B. (2020). HLPpred-Fuse: Improved and Robust Prediction of Hemolytic Peptide and its Activity by Fusing Multiple Feature Representation. Bioinformatics (Oxford, England) 36 (11), 3350–3356. 10.1093/bioinformatics/btaa160 32145017

[B32] He S. Guo F. Zou Q. HuiDing H. (2021). MRMD2.0: A Python Tool for Machine Learning with Feature Ranking and Reduction. Cbio 15 (10), 1213–1221. 10.2174/1574893615999200503030350

[B33] Hoang D. T. Chernomor O. von Haeseler A. Minh B. Q. Vinh L. S. (2018). UFBoot2: Improving the Ultrafast Bootstrap Approximation. Mol. Biol. Evol. 35 (2), 518–522. 10.1093/molbev/msx281 29077904 PMC5850222

[B34] Hong Z. Zeng X. Wei L. Liu X. (2019). Identifying Enhancer-Promoter Interactions with Neural Network Based on Pre-trained DNA Vectors and Attention Mechanism. Bioinformatics 36 (4), 1037–1043. 10.1093/bioinformatics/btz694 31588505

[B35] Huang Y. Zhou D. Wang Y. Zhang X. Su M. Wang C. (2020). Prediction of Transcription Factors Binding Events Based on Epigenetic Modifications in Different Human Cells. Epigenomics 12 (16), 1443–1456. 10.2217/epi-2019-0321 32921165

[B36] Jensen H. H. Login F. H. Koffman J. S. Kwon T.-H. Nejsum L. N. (2016). The Role of Aquaporin-5 in Cancer Cell Migration: A Potential Active Participant. Int. J. Biochem. Cell Biol. 79, 271–276. 10.1016/j.biocel.2016.09.005 27609140

[B37] Jiang Q. Wang G. Jin S. Li Y. Wang Y. (2013). Predicting Human microRNA-Disease Associations Based on Support Vector Machine. Ijdmb 8 (3), 282–293. 10.1504/ijdmb.2013.056078 24417022

[B38] Jin Q. Cui H. Sun C. Meng Z. Su R. (2021). Free-form Tumor Synthesis in Computed Tomography Images via Richer Generative Adversarial Network. Knowledge-Based Syst. 218, 106753. 10.1016/j.knosys.2021.106753

[B39] Jin S. Zeng X. Xia F. Huang W. Liu X. (2020). Application of Deep Learning Methods in Biological Networks. Brief Bioinform 22 (2), 1902–1917. 10.1093/bib/bbaa043 32363401

[B40] Jung H. J. Park J.-Y. Jeon H.-S. Kwon T.-H. (2011). Aquaporin-5: a Marker Protein for Proliferation and Migration of Human Breast Cancer Cells. PLoS One 6 (12), e28492. 10.1371/journal.pone.0028492 22145049 PMC3228775

[B41] Jung Y. Zhang H. Hu J. (2019). Transformed Low-Rank ANOVA Models for High-Dimensional Variable Selection. Stat. Methods Med. Res. 28 (4), 1230–1246. 10.1177/0962280217753726 29384042

[B42] Kalyaanamoorthy S. Minh B. Q. Wong T. K. F. von Haeseler A. Jermiin L. S. (2017). ModelFinder: Fast Model Selection for Accurate Phylogenetic Estimates. Nat. Methods 14 (6), 587–589. 10.1038/nmeth.4285 28481363 PMC5453245

[B43] Kang S. K. Chae Y. K. Woo J. Kim M. S. Park J. C. Lee J. (2008). Role of Human Aquaporin 5 in Colorectal Carcinogenesis. Am. J. Pathol. 173 (2), 518–525. 10.2353/ajpath.2008.071198 18583321 PMC2475788

[B44] Kasa P. Farran B. Prasad G. L. V. Nagaraju G. P. (2019). Aquaporins in Female Specific Cancers. Gene 700, 60–64. 10.1016/j.gene.2019.03.032 30898710

[B45] Kröger S. Wolburg H. Warth A. (2004). Redistribution of Aquaporin-4 in Human Glioblastoma Correlates with Loss of Agrin Immunoreactivity from Brain Capillary Basal Laminae. Acta neuropathologica 107 (4), 311–318. 10.1007/s00401-003-0812-0 14735305

[B46] Kumar M. Gromiha M. M. Raghava G. P. (2007). Identification of DNA-Binding Proteins Using Support Vector Machines and Evolutionary Profiles. BMC Bioinformatics 8, 463. 10.1186/1471-2105-8-463 18042272 PMC2216048

[B47] Lai H.-Y. Zhang Z.-Y. Su Z.-D. Su W. Ding H. Chen W. (2019). iProEP: A Computational Predictor for Predicting Promoter. Mol. Ther. - Nucleic Acids 17, 337–346. 10.1016/j.omtn.2019.05.028 31299595 PMC6616480

[B48] Lan Y.-L. Wang X. Lou J.-C. Ma X.-C. Zhang B. (2017). The Potential Roles of Aquaporin 4 in Malignant Gliomas. Oncotarget 8 (19), 32345–32355. 10.18632/oncotarget.16017 28423683 PMC5458289

[B49] Lee S. J. Chae Y. S. Kim J. G. Kim W. W. Jung J. H. Park H. Y. (2014). AQP5 Expression Predicts Survival in Patients with Early Breast Cancer. Ann. Surg. Oncol. 21 (2), 375–383. 10.1245/s10434-013-3317-7 24114055

[B50] Levin M. H. Verkman A. S. (2006). Aquaporin-3-dependent Cell Migration and Proliferation during Corneal Re-epithelialization. Invest. Ophthalmol. Vis. Sci. 47 (10), 4365–4372. 10.1167/iovs.06-0335 17003427

[B51] Li J. Pu Y. Tang J. Zou Q. Guo F. (2020). DeepATT: a Hybrid Category Attention Neural Network for Identifying Functional Effects of DNA Sequences. Brief. Bioinform. 22, 1. 10.1093/bib/bbaa159 32778871

[B52] Li M. Xu H. Deng Y. (2019). Evidential Decision Tree Based on Belief Entropy. Entropy 21 (9), 897. 10.3390/e21090897

[B53] Li Y. Niu M. Zou Q. Elm-M. H. C. (2019). ELM-MHC: An Improved MHC Identification Method with Extreme Learning Machine Algorithm. J. Proteome Res. 18 (3), 1392–1401. 10.1021/acs.jproteome.9b00012 30698979

[B54] Lin C.-W. Chen P.-N. Chen M.-K. Yang W.-E. Tang C.-H. Yang S.-F. (2013). Kaempferol Reduces Matrix Metalloproteinase-2 Expression by Down-Regulating ERK1/2 and the Activator Protein-1 Signaling Pathways in Oral Cancer Cells. PLoS One 8 (11), e80883. 10.1371/journal.pone.0080883 24278338 PMC3835430

[B55] Lin H. Liang Z.-Y. Tang H. Chen W. (2019). Identifying Sigma70 Promoters with Novel Pseudo Nucleotide Composition. Ieee/acm Trans. Comput. Biol. Bioinf. 16 (4), 1316–1321. 10.1109/tcbb.2017.2666141 28186907

[B56] Liu B. Gao X. Zhang H. (2019). BioSeq-Analysis2.0: an Updated Platform for Analyzing DNA, RNA and Protein Sequences at Sequence Level and Residue Level Based on Machine Learning Approaches. Nucleic Acids Res. 47 (20), e127. 10.1093/nar/gkz740 31504851 PMC6847461

[B57] Liu B. Xu J. Lan X. Xu R. Zhou J. Wang X. (2014). iDNA-Prot|dis: Identifying DNA-Binding Proteins by Incorporating Amino Acid Distance-Pairs and Reduced Alphabet Profile into the General Pseudo Amino Acid Composition. PLoS One 9 (9), e106691. 10.1371/journal.pone.0106691 25184541 PMC4153653

[B58] Liu B. Zhu Y. Yan K. (2020). Fold-LTR-TCP: Protein Fold Recognition Based on Triadic Closure Principle. Brief. Bioinform. 21 (6), 2185–2193. 10.1093/bib/bbz139 31813954

[B59] Liu J. Su R. Zhang J. Wei L. (2021). Classification and Gene Selection of Triple-Negative Breast Cancer Subtype Embedding Gene Connectivity Matrix in Deep Neural Network. Brief Bioinform 22, bbaa395. 10.1093/bib/bbaa395 33415328

[B60] Liu M.-L. Su W. Wang J.-S. Yang Y.-H. Yang H. Lin H. (2020). Predicting Preference of Transcription Factors for Methylated DNA Using Sequence Information. Mol. Ther. - Nucleic Acids 22, 1043–1050. 10.1016/j.omtn.2020.07.035 33294291 PMC7691157

[B61] Liu X.-J. Gong X.-J. Yu H. Xu J.-H. (2018). A Model Stacking Framework for Identifying DNA Binding Proteins by Orchestrating Multi-View Features and Classifiers. Genes 9 (8), 394. 10.3390/genes9080394 PMC6116045 30071697

[B62] Liu Y. Ouyang X.-h. Xiao Z.-X. Zhang L. Cao Y. (2021). A Review on the Methods of Peptide-MHC Binding Prediction. Cbio 15 (8), 878–888. 10.2174/1574893615999200429122801

[B63] Lou W. Wang X. Chen F. Chen Y. Jiang B. Zhang H. (2014). Sequence Based Prediction of DNA-Binding Proteins Based on Hybrid Feature Selection Using Random Forest and Gaussian Naïve Bayes. PLoS One 9 (1), e86703. 10.1371/journal.pone.0086703 24475169 PMC3901691

[B64] Luche S. Santoni V. Rabilloud T. (2003). Evaluation of Nonionic and Zwitterionic Detergents as Membrane Protein Solubilizers in Two-Dimensional Electrophoresis. Proteomics 3 (3), 249–253. 10.1002/pmic.200390037 12627377

[B65] Ma T. Yang B. Verkman A. S. (1997). Cloning of a Novel Water and Urea-Permeable Aquaporin from Mouse Expressed Strongly in colon, Placenta, Liver, and Heart. Biochem. Biophysical Res. Commun. 240 (2), 324–328. 10.1006/bbrc.1997.7664 9388476

[B66] Manavalan B. Basith S. Shin T. H. Wei L. Lee G. (2019). mAHTPred: a Sequence-Based Meta-Predictor for Improving the Prediction of Anti-hypertensive Peptides Using Effective Feature Representation. Bioinformatics 35 (16), 2757–2765. 10.1093/bioinformatics/bty1047 30590410

[B67] Manavalan B. Basith S. Shin T. H. Wei L. Lee G. (2019). Meta-4mCpred: A Sequence-Based Meta-Predictor for Accurate DNA 4mC Site Prediction Using Effective Feature Representation. Mol. Ther. - Nucleic Acids 16, 733–744. 10.1016/j.omtn.2019.04.019 31146255 PMC6540332

[B68] Marlar S. Jensen H. H. Login F. H. Nejsum L. N. (2017). Aquaporin-3 in Cancer. Ijms 18 (10), 2106. 10.3390/ijms18102106 PMC5666788 28991174

[B69] Maugeri R. Schiera G. Di Liegro C. Fricano A. Iacopino D. Di Liegro I. (2016). Aquaporins and Brain Tumors. Ijms 17 (7), 1029. 10.3390/ijms17071029 PMC4964405 27367682

[B70] Minh B. Q. Nguyen M. A. T. von Haeseler A. (2013). Ultrafast Approximation for Phylogenetic Bootstrap. Mol. Biol. Evol. 30 (5), 1188–1195. 10.1093/molbev/mst024 23418397 PMC3670741

[B71] Mobasheri A. Airley R. Hewitt S. Marples D. (2005). Heterogeneous Expression of the Aquaporin 1 (AQP1) Water Channel in Tumors of the Prostate, Breast, Ovary, colon and Lung: a Study Using High Density Multiple Human Tumor Tissue Microarrays. Int. J. Oncol. 26 (5), 1149–1158. 10.3892/ijo.26.5.1149 15809704

[B72] Moon C. Soria J.-C. Jang S. J. Lee J. Hoque M. O. Sibony M. (2003). Involvement of Aquaporins in Colorectal Carcinogenesis. Oncogene 22 (43), 6699–6703. 10.1038/sj.onc.1206762 14555983

[B73] Muhammod R. Ahmed S. Md Farid D. Shatabda S. Sharma A. Dehzangi A. (2019). PyFeat: a Python-Based Effective Feature Generation Tool for DNA, RNA and Protein Sequences. Bioinformatics (Oxford, England) 35 (19), 3831–3833. 10.1093/bioinformatics/btz165 PMC6761934 30850831

[B74] Nagaraju G. P. Basha R. Rajitha B. Alese O. B. Alam A. Pattnaik S. (2016). Aquaporins: Their Role in Gastrointestinal Malignancies. Cancer Lett. 373 (1), 12–18. 10.1016/j.canlet.2016.01.003 26780474

[B75] Nakahigashi K. Kabashima K. Ikoma A. Verkman A. S. Miyachi Y. Hara-Chikuma M. (2011). Upregulation of Aquaporin-3 Is Involved in Keratinocyte Proliferation and Epidermal Hyperplasia. J. Invest. Dermatol. 131 (4), 865–873. 10.1038/jid.2010.395 21191421

[B76] Nielsen S. Frøkiær J. Marples D. Kwon T.-H. Agre P. Knepper M. A. (2002). Aquaporins in the Kidney: from Molecules to Medicine. Physiol. Rev. 82 (1), 205–244. 10.1152/physrev.00024.2001 11773613

[B77] Park E. J. Jung H. J. Choi H. J. Jang H. J. Park H. J. Nejsum L. N. (2020). Exosomes Co‐expressing AQP5‐targeting miRNAs and IL‐4 Receptor‐binding Peptide Inhibit the Migration of Human Breast Cancer Cells. FASEB j. 34 (2), 3379–3398. 10.1096/fj.201902434R 31922312

[B78] Preston G. M. Carroll T. P. Guggino W. B. Agre P. (1992). Appearance of Water Channels in Xenopus Oocytes Expressing Red Cell CHIP28 Protein. Science 256 (5055), 385–387. 10.1126/science.256.5055.385 1373524

[B79] Rawlings A. E. (2016). Membrane Proteins: Always an Insoluble Problem? Biochem. Soc. Trans. 44, 790–795. 10.1042/BST20160025 27284043 PMC4900757

[B80] Rojek A. Praetorius J. Frøkiaer J. Nielsen S. Fenton R. A. (2008). A Current View of the Mammalian Aquaglyceroporins. Annu. Rev. Physiol. 70, 301–327. 10.1146/annurev.physiol.70.113006.100452 17961083

[B81] Ru X. Li L. Zou Q. (2019). Incorporating Distance-Based Top-N-Gram and Random Forest to Identify Electron Transport Proteins. J. Proteome Res. 18 (7), 2931–2939. 10.1021/acs.jproteome.9b00250 31136183

[B82] Saadoun S. Papadopoulos M. C. Davies D. C. Krishna S. Bell B. A. (2002). Aquaporin-4 Expression Is Increased in Oedematous Human Brain Tumours. J. Neurol. Neurosurg. Psychiatry 72 (2), 262–265. 10.1136/jnnp.72.2.262 11796780 PMC1737753

[B83] Saadoun S. Papadopoulos M. C. Davies D. C. Bell B. A. Krishna S. (2002). Increased Aquaporin 1 Water Channel Expression Inhuman Brain Tumours. Br. J. Cancer 87 (6), 621–623. 10.1038/sj.bjc.6600512 12237771 PMC2364235

[B84] Saadoun S. Papadopoulos M. C. Hara-Chikuma M. Verkman A. S. (2005). Impairment of Angiogenesis and Cell Migration by Targeted Aquaporin-1 Gene Disruption. Nature 434 (7034), 786–792. 10.1038/nature03460 15815633

[B85] Saadoun S. Papadopoulos M. C. Watanabe H. Yan D. Manley G. T. Verkman A. S. (2005). Involvement of Aquaporin-4 in Astroglial Cell Migration and Glial Scar Formation. J. Cel. Sci. 118 (Pt 24), 5691–5698. 10.1242/jcs.02680 16303850

[B86] Shanahan H. P. Garcia M. A. Jones S. Thornton J. M. (2004). Identifying DNA-Binding Proteins Using Structural Motifs and the Electrostatic Potential. Nucleic Acids Res. 32 (16), 4732–4741. 10.1093/nar/gkh803 15356290 PMC519102

[B87] Shao J. Liu B. (2021). ProtFold-DFG: Protein Fold Recognition by Combining Directed Fusion Graph and PageRank Algorithm. Brief Bioinform 22 (3), bbaa192. 10.1093/bib/bbaa192 32892224

[B88] Shao J. Yan K. Liu B. (2021). FoldRec-C2C: Protein Fold Recognition by Combining Cluster-To-Cluster Model and Protein Similarity Network. Brief Bioinform 22 (3), bbaa144. 10.1093/bib/bbaa144 32685972 PMC7454262

[B89] Shen Z. Zou Q. (2020). Basic Polar and Hydrophobic Properties Are the Main Characteristics that Affect the Binding of Transcription Factors to Methylation Sites. Bioinformatics 36 (15), 4263–4268. 10.1093/bioinformatics/btaa492 32399547

[B90] Su R. Hu J. Zou Q. Manavalan B. Wei L. (2020). Empirical Comparison and Analysis of Web-Based Cell-Penetrating Peptide Prediction Tools. Brief. Bioinform. 21 (2), 408–420. 10.1093/bib/bby124 30649170

[B91] Su R. Liu X. Wei L. Zou Q. (2019). Deep-Resp-Forest: A Deep forest Model to Predict Anti-cancer Drug Response. Methods 166, 91–102. 10.1016/j.ymeth.2019.02.009 30772464

[B92] Su R. Wu H. Xu B. Liu X. Wei L. (2019). Developing a Multi-Dose Computational Model for Drug-Induced Hepatotoxicity Prediction Based on Toxicogenomics Data. Ieee/acm Trans. Comput. Biol. Bioinf. 16 (4), 1231–1239. 10.1109/tcbb.2018.2858756 30040651

[B93] Su W. Liu M.-L. Yang Y.-H. Wang J.-S. Li S.-H. Lv H. (2021). PPD: A Manually Curated Database for Experimentally Verified Prokaryotic Promoters. J. Mol. Biol. 433 (11), 166860. 10.1016/j.jmb.2021.166860 33539888

[B94] Szilágyi A. Skolnick J. (2006). Efficient Prediction of Nucleic Acid Binding Function from Low-Resolution Protein Structures. J. Mol. Biol. 358 (3), 922–933. 10.1016/j.jmb.2006.02.053 16551468

[B95] Tang H. Zhao Y.-W. Zou P. Zhang C.-M. Chen R. Huang P. (2018). HBPred: a Tool to Identify Growth Hormone-Binding Proteins. Int. J. Biol. Sci. 14 (8), 957–964. 10.7150/ijbs.24174 29989085 PMC6036759

[B96] Tang Y.-J. Pang Y.-H. Liu B. Idp-Seq2Seq (2020). IDP-Seq2Seq: Identification of Intrinsically Disordered Regions Based on Sequence to Sequence Learning. Bioinformaitcs 36 (21), 5177–5186. 10.1093/bioinformatics/btaa667 32702119

[B97] Tyagi A. Kapoor P. Kumar R. Chaudhary K. Gautam A. Raghava G. P. S. (2013). In Silico models for Designing and Discovering Novel Anticancer Peptides. Sci. Rep. 3, 2984. 10.1038/srep02984 24136089 PMC6505669

[B98] Verkman A. S. (2005). More Than Just Water Channels: Unexpected Cellular Roles of Aquaporins. J. Cel. Sci. 118 (Pt 15), 3225–3232. 10.1242/jcs.02519 16079275

[B99] Wang D. Owler B. K. (2011). Expression of AQP1 and AQP4 in Paediatric Brain Tumours. J. Clin. Neurosci. 18 (1), 122–127. 10.1016/j.jocn.2010.07.115 20965731

[B100] Wang H. Ding Y. Tang J. Guo F. (2020). Identification of Membrane Protein Types via Multivariate Information Fusion with Hilbert-Schmidt Independence Criterion. Neurocomputing 383, 257–269. 10.1016/j.neucom.2019.11.103

[B101] Warth A. Simon P. Capper D. Goeppert B. Tabatabai G. Herzog H. (2007). Expression Pattern of the Water Channel Aquaporin-4 in Human Gliomas Is Associated with Blood-Brain Barrier Disturbance but Not with Patient Survival. J. Neurosci. Res. 85 (6), 1336–1346. 10.1002/jnr.21224 17335082

[B102] Wei L. Chen H. Su R. (2018). M6APred-EL: A Sequence-Based Predictor for Identifying N6-Methyladenosine Sites Using Ensemble Learning. Mol. Ther. - Nucleic Acids 12, 635–644. 10.1016/j.omtn.2018.07.004 30081234 PMC6082921

[B103] Wei L. Hu J. Li F. Song J. Su R. Zou Q. (2020). Comparative Analysis and Prediction of Quorum-sensing Peptides Using Feature Representation Learning and Machine Learning Algorithms. Brief. Bioinform. 21 (1), 106–119. 10.1093/bib/bby107 30383239

[B104] Wei L. Liao M. Gao Y. Ji R. He Z. Zou Q. (2014). Improved and Promising Identification of Human MicroRNAs by Incorporating a High-Quality Negative Set. Ieee/acm Trans. Comput. Biol. Bioinf. 11 (1), 192–201. 10.1109/tcbb.2013.146 26355518

[B105] Wei L. Tang J. Zou Q. (2017). Local-DPP: An Improved DNA-Binding Protein Prediction Method by Exploring Local Evolutionary Information. Inf. Sci. 384, 135–144. 10.1016/j.ins.2016.06.026

[B106] Wei L. Wan S. Guo J. Wong K. K. (2017). A Novel Hierarchical Selective Ensemble Classifier with Bioinformatics Application. Artif. Intelligence Med. 83, 82–90. 10.1016/j.artmed.2017.02.005 28245947

[B107] Wei L. Xing P. Zeng J. Chen J. Su R. Guo F. (2017). Improved Prediction of Protein-Protein Interactions Using Novel Negative Samples, Features, and an Ensemble Classifier. Artif. Intelligence Med. 83, 67–74. 10.1016/j.artmed.2017.03.001 28320624

[B108] Wei L. Zhou C. Chen H. Song J. Su R. (2018). ACPred-FL: a Sequence-Based Predictor Using Effective Feature Representation to Improve the Prediction of Anti-cancer Peptides. Bioinformatics 34 (23), 4007–4016. 10.1093/bioinformatics/bty451 29868903 PMC6247924

[B109] Wu X. Yu L. (2021). EPSOL: Sequence-Based Protein Solubility Prediction Using Multidimensional Embedding. Bioinformatics (Oxford, England) 37, 4314–4320. 10.1093/bioinformatics/btab463 34145885

[B110] Yang H. Luo Y. Ren X. Wu M. He X. Peng B. (2021). Risk Prediction of Diabetes: Big Data Mining with Fusion of Multifarious Physical Examination Indicators. Inf. Fusion 75, 140–149. 10.1016/j.inffus.2021.02.015

[B111] Yu L. Wang M. Yang Y. Xu F. Zhang X. Xie F. (2021). Predicting Therapeutic Drugs for Hepatocellular Carcinoma Based on Tissue-specific Pathways. Plos Comput. Biol. 17 (2), e1008696. 10.1371/journal.pcbi.1008696 33561121 PMC7920387

[B112] Yu X. Zhou J. Zhao M. Yi C. Duan Q. Zhou W. (2021). Exploiting XG Boost for Predicting Enhancer-Promoter Interactions. Cbio 15 (9), 1036–1045. 10.2174/1574893615666200120103948

[B113] Zeng X. Liao Y. Liu Y. Zou Q. (2017). Prediction and Validation of Disease Genes Using HeteSim Scores. Ieee/acm Trans. Comput. Biol. Bioinf. 14 (3), 687–695. 10.1109/tcbb.2016.2520947 26890920

[B114] Zeng X. Liu L. Lü L. Zou Q. (2018). Prediction of Potential Disease-Associated microRNAs Using Structural Perturbation Method. Bioinformatics 34 (14), 2425–2432. 10.1093/bioinformatics/bty112 29490018

[B115] Zeng X. Zhu S. Liu X. Zhou Y. Nussinov R. Cheng F. (2019). deepDR: a Network-Based Deep Learning Approach to In Silico Drug Repositioning. Bioinformatics 35 (24), 5191–5198. 10.1093/bioinformatics/btz418 31116390 PMC6954645

[B116] Zeng X. Zhu S. Lu W. Liu Z. Huang J. Zhou Y. (2020). Target Identification Among Known Drugs by Deep Learning from Heterogeneous Networks. Chem. Sci. 11 (7), 1775–1797. 10.1039/c9sc04336e 34123272 PMC8150105

[B117] Zhang D. Chen H.-D. Zulfiqar H. Yuan S.-S. Huang Q.-L. Zhang Z.-Y. (2021). iBLP: An XGBoost-Based Predictor for Identifying Bioluminescent Proteins. Comput. Math. Methods Med. 2021, 1–15. 10.1155/2021/6664362 PMC7808816 33505515

[B118] Zhang J. Chen Q. Liu B. (2020). iDRBP_MMC: Identifying DNA-Binding Proteins and RNA-Binding Proteins Based on Multi-Label Learning Model and Motif-Based Convolutional Neural Network. J. Mol. Biol. 432 (22), 5860–5875. 10.1016/j.jmb.2020.09.008 32920048

[B119] Zhang L. Xiao X. Xu Z.-C. (2020). iPromoter-5mC: A Novel Fusion Decision Predictor for the Identification of 5-Methylcytosine Sites in Genome-wide DNA Promoters. Front. Cell Dev. Biol. 8, 614. 10.3389/fcell.2020.00614 32850787 PMC7399635

[B120] Zhang Z. Chen Z. Song Y. Zhang P. Hu J. Bai C. (2010). Expression of Aquaporin 5 Increases Proliferation and Metastasis Potential of Lung Cancer. J. Pathol. 221 (2), 210–220. 10.1002/path.2702 20455256

[B121] Zhu X.-J. Feng C.-Q. Lai H.-Y. Chen W. Hao L. (2019). Predicting Protein Structural Classes for Low-Similarity Sequences by Evaluating Different Features. Knowledge-Based Syst. 163, 787–793. 10.1016/j.knosys.2018.10.007

[B122] Zhu Y. Li F. Xiang D. Akutsu T. Song J. Jia C. (2021). Computational Identification of Eukaryotic Promoters Based on Cascaded Deep Capsule Neural Networks. Brief Bioinform 22 (4), bbaa299. 10.1093/bib/bbaa299 33227813 PMC8522485

[B123] Zou Q. Lin G. Jiang X. Liu X. Zeng X. (2020). Sequence Clustering in Bioinformatics: an Empirical Study. Brief. Bioinform. 21 (1), 1–10. 10.1093/bib/bby090 30239587

